# Severe Radial Artery Spasm Causing Radial Artery Sheath Entrapment: A Case Report

**DOI:** 10.7759/cureus.80161

**Published:** 2025-03-06

**Authors:** Zahid Khan, Tat Koh

**Affiliations:** 1 Acute Medicine, Mid and South Essex NHS Foundation Trust, Southend-on-Sea, GBR; 2 Cardiology, Bart’s Heart Centre UK, London, GBR; 3 Cardiology and General Medicine, Barking, Havering and Redbridge University Hospitals NHS Trust, London, GBR; 4 Cardiology, Royal Free Hospital, London, GBR; 5 Cardiology, Bart's Heart Centre, London, GBR

**Keywords:** clamp and release technique, coronary artery angiogram, intratracheal intubation, iv propofol, radial artery spasm, radial endarterectomy, regional nerve block, retained radial artery sheath, vasodilators, warm compressors

## Abstract

Over the past decade, there has been a significant increase in the use of the transradial approach in patients undergoing coronary angiography due to lower risk of complications and quicker recovery. Nevertheless, radial artery spasm remains a challenge, particularly in anxious men and women, and young female patients. Transradial access is associated with a significantly lower bleeding risk compared to transfemoral access. The radial artery is predisposed to significant spasms as it has a smaller caliber and is highly sensitive to mechanical and humoral stresses compared to femoral artery. We present the case of an 82-year-old male who was transferred from a district general hospital for coronary angiography and developed severe spasm in the right radial artery at the end of the procedure, resulting in radial artery sheath entrapment. We administered 600 microgram of intra-arterial nitrates, 2.5 mg intra-arterial verapamil, 3.5 mg midazolam, and 2.5 mg intravenous morphine with no effect on the spasm. Following this, we applied a blood pressure cuff and inflated it to 156 mmHg, which was 40 mmHg greater than his systolic blood pressure, for five minutes to occlude the brachial artery. This resulted in smooth muscle relaxation facilitating the removal of radial sheath without any adverse outcomes.

## Introduction

The transradial approach (TRA) has gained traction and is preferred over the transfemoral approach (TFA) because of the lower risk of complications in patients undergoing coronary angioplasties. Historically, the transfemoral approach has been the main approach for coronary interventions; however, with technological advancement, this has changed, and transradial or transulnar approaches are preferred [1]. The use of the TRA varies significantly, ranging from 16.1% in the United States to 73% in Italy [2,3]. The lesser familiarity is one of the main reasons for lower use of the TRA for coronary angioplaties in the United States [3]. The transradial approach is further supported by the use of vascular ultrasound imaging in patients with weaker radial pulses. However, this variation in the use of the TRA is associated with limited learning opportunities and more complications in the hands of inexperienced interventionists [1]. Several complications are associated with TRA, including radial artery spasm and increased fluoroscopy time, and patients may receive a larger radiation dose than during a TFA [1]. 

Radial artery spasm is defined as the sudden constriction and narrowing of the radial artery, which can result in procedural failure due to the inability to advance the catheter. This is reported in approximately 30% of patients undergoing coronary intervention [4,5]. Patient demographic factors, such as female sex, increasing age, shorter patients, and low body mass index (BMI) are associated with an increased risk of RAS. Radial artery anatomy, procedural factors (weak pulse, multiple radial puncture attempts, insertion of a ≥ 7F sheath) and cardiovascular risk factors, including hypertension, smoking, and anxiety, are associated with an increased risk of RAS [1,6,7]. Based on these risk factors, a predictive score has been developed that includes BMI, hypertension, smoking status, height, and peripheral artery disease [7,8]. This score enables the implementation of strategies to minimise the risk of RAS. Based on this scoring system, our patient had a history of smoking and hypertension as risk factors. We present a case of an 82-year-old male who underwent coronary angiogram via right radial access and developed severe spasm post-procedure, resulting in radial sheath entrapment. 

## Case presentation

An 82-year-old man was referred from the cardiology outpatient clinic for suspected angina. He was a tall (187cm) and slim (71kg) elderly patient. His past medical history was significant for primary coronary angioplasty with a single stent in the right coronary artery (RCA) for ST-elevated myocardial infarction (STEMI) in 2020, atrial fibrillation during the STEMI presentation, gastroesophageal reflux disease, and recent prostate surgery followed by haematuria for 2 weeks. Regular medications included rivaroxaban 20 mg once daily (OD), lansoprazole 30 mg OD, gaviscon pro re nata (PRN), bisoprolol, famotidine, simvsatatin due to intolerance to atorvastatin and rosuvastatin, solifenacin, latanoprost eye drops, rampiril, and glyeryl trinitrate spray (PRN). A myocardial perfusion scan in 2022 showed normal left ventricular systolic function with a left ventricular ejection fraction (LVEF) of 61% and no evidence of inducible myocardial ischaemia. A partial-thickness inferior infarction extended into the basal inferoseptal region and preserved viability elsewhere.

Due to a lack of a radial pulse in the right wrist, a left-sided radial approach was used to perform the coronary angiography with right femoral access as back up if the left radial access failed. We used a standard 6Fr radial sheath and two attempts were made to obtain left radial access. Both punctures were successful; however, as we were unable to feed the wire through the first distal radial puncture, a more proximal second puncture was made using a terumo needle. The patient received 2.5 mg verapamil intra-arterially through the left radial sheath, and midazolam 0.5 mg intravenously. He also received 5000 units intraarterial heparin into the aortic root during the coronary angiogram. The coronary angiogram was performed using standard 6Fr Judkins diagnostic catheters, JR4 and JL3.5. The coronary angiogram demonstrated a patent RCA stent, mild disease in the mid vessel distal to the stent, and mild diffuse disease in the left-sided arteries (Videos 1-2). 

**Video 1:** Coronary angiogram of the right coronary artery showing patent stent and mild mid vessel narrowing.

**Video 2:** Coronary angiogram of the left coronary artery showing mild diffuse disease in the distal left anterior descending and left circumflex arteries.

Following the coronary angiogram, the patient developed a severe spasm in the left radial artery, leading to entrapment of the radial sheath as we could not remove the sheath. He was given 1 mg midazolam and 2.5 mg morphine intravenously to relax him in order to remove the sheath. As this did not yield any results, an additional 1 mg of intravenous midazolam and 2.5 mg of intra-arterial verapamil were administered. The patient continued to have spasms in his left radial artery and a further 1.5 mg midazolam and 400 µg intra-arterial nitrate was administered without any significant result. Following this, the patient became quite drowsy; however, he remained arousable. At this point, anaesthetic support was requested anticipating that the patient may require deep sedation using propofol or general anesthatics in order to remove the radial sheath. In the presence of the anaesthetist, we then applied a manual blood pressure cuff around the left arm inflating it to 156 mmHg (40 mmHg higher than his systolic blood pressure of 116 mmHg) for 5 minutes, followed by quick release known as "clamp-and-release" technique. This technique leads to peripheral ischaemia and smooth muscle relaxation resulting in arterial vasodilatation, and allowed us to remove the radial sheath without any complications. As the patient was slightly drowsy following the administration of the abovementioned medications, he was monitored for a few hours post procedure and was discharged home later the same day. He was advised to increase the dose of lansoprazole to 30 mg twice daily because his chest pain was suspected to be caused by gastroesophageal reflux disease. As our patient had an intact and palpable left radial pulse several hours after the removal of entrapped radial sheath, no further imaging was performed. In view of the non-cardiac history of chest pain, further cardiac investigations were not performed. 

## Discussion

The transradial approach is becoming increasingly common in patients undergoing coronary angioplasty; however, radial artery spasm (RAS) remains a major challenge. The prevalence of RAS is reported to be 14.2% in the literature, and a cross-sectional study in Australia based on 169 patients has shown a similar prevalance [1]. Curtis et al. reported radial access complications in 32 patients and RAS was observed in 24 (14.2%) patients. Additional complications include radial artery dissection, pseudoaneurysm, infection, and brachial perforation. There was no significant difference in the rates of RAS between procedures carried out by the trainees or consultant cardiologists, and crossover to the femoral artery was required in eight (4.7%) patients, five of which were due to RAS [1]. Univariate analysis demonstrated a significantly higher incidence of RAS in patients with a history of anxiety, female sex, increased length of procedure time, and patients younger than 65 years. Most patients in this study received midazolam, nitrates, and fentanyl to avoid RAS, and only five patients did not receive any medications before the procedure. There were no statistically significant differences in the incidence of RAS or medication use. 

Feldman et al. published their report based on data from the National Cardiovascular Registry of patients undergoing PCI between 2007 and 2012 [2]. This study demonstrated an increase in the proportion of radial access from 1.2% in the first quarter of 2007 to 16.1% in the third quarter of 2012, accounting for 6.3% of the total procedures from 2007 to 2012. The study showed that younger patients, women, and patients with a higher body mass index (BMI) were more likely to undergo the procedure performed through radial access. These patients also had a lower prevalence of renal insufficiency, myocardial infarction, bypass surgery or PCI, congestive heart failure, and peripheral vascular disease. The fluoroscopy time was 14.2 minutes for patients undergoing coronary angioplasty via radial access, compared to 11.1 minutes for those using femoral access. The amount of contrast used was slightly less in radial access PCI than in femoral access PCI. Another rare complication of TRA is radial artery avulsion, which may require endovascular repair [9]. Nitrates cause smooth muscle relaxation, leading to vasodilatation. Verapamil blocks calcium channels in the smooth muscle cells, preventing calcium influx which is necessary for muscle contraction, resulting in vasodilation. Opioid and benzodiazipine sedatives, such as morphine and midazolam, decrease discomfort and anxiety in patients, which may help reduce the stimulation of neural pathways and arterial vasoconstriction [1]. The clamp-and-release technique causes ischaemia, resulting in smooth muscle relaxation and relieving the spasm. 

The Society for Cardiovascular Angiography and Interventions (SCAI) published stepwise guidelines for managing radial artery sheath entrapment [10]. These guidelines reported the incidence of RAS to be approximately 15-30%, ranging from mild to severe. Radial artery spasm occurs primarily due to the high muscular media and density of alpha receptors in the radial artery, and severe refractory spasms can result in radial sheath entrapment. The incidence of severe RAS was reported to be 0.7% during diagnostic procedures, and 1.3% during therapeutic procedures by Zencirci et al. [10]. Multiple arterial punctures, long procedural duration, large-bore sheaths, use of numerous exchange catheters, and patient anxiety were significant predictors of moderate-to-severe spasms. Forceful removal of the radial artery sheath in patients with RAS can lead to avulsion of the radial artery and may result in endarterectomy [10]. Schenke et al. enrolled 327 patients undergoing coronary angioplasty via transradial acccess in the TRIANGLE Registry (TwitteR Initiated registry for coronary ANgiography in Germany via distaL radial accEss) [11]. This study reported that < 1% of patients had distal radial artery occlusion on ultrasound screening and the puncture time was less in patients with distal right radial access compared to the left. SImilarly, longer fluoroscopy time and contrast use was more in patients undergoing coronary angioplasty via left distal radial artery compared to right distal radial artery [11]. 

**Figure 1:**
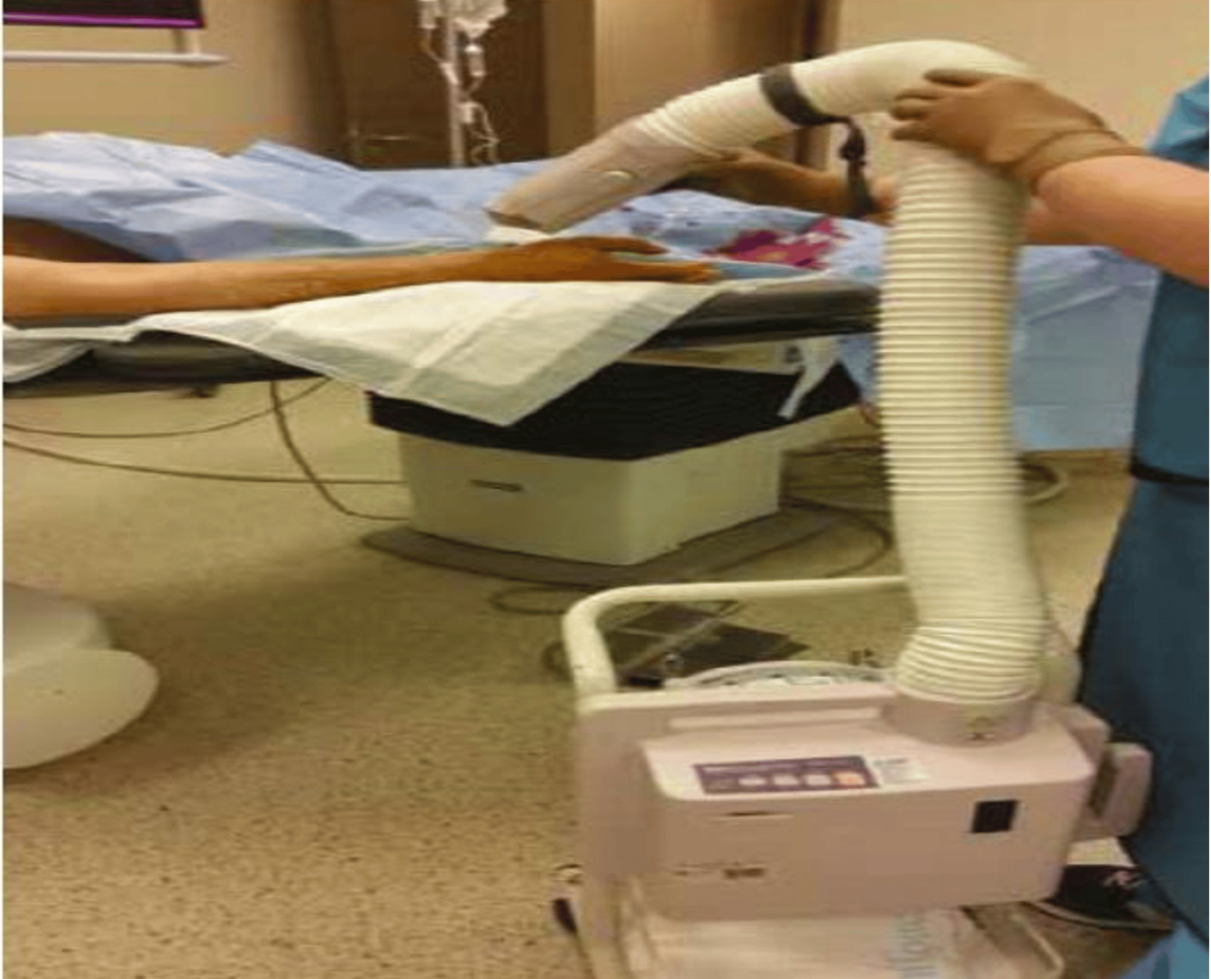
Forearm warming techniques with a convective air patient warming system Reproduced with permission from Zencirci et al., 2016 [10].

**Figure 2:**
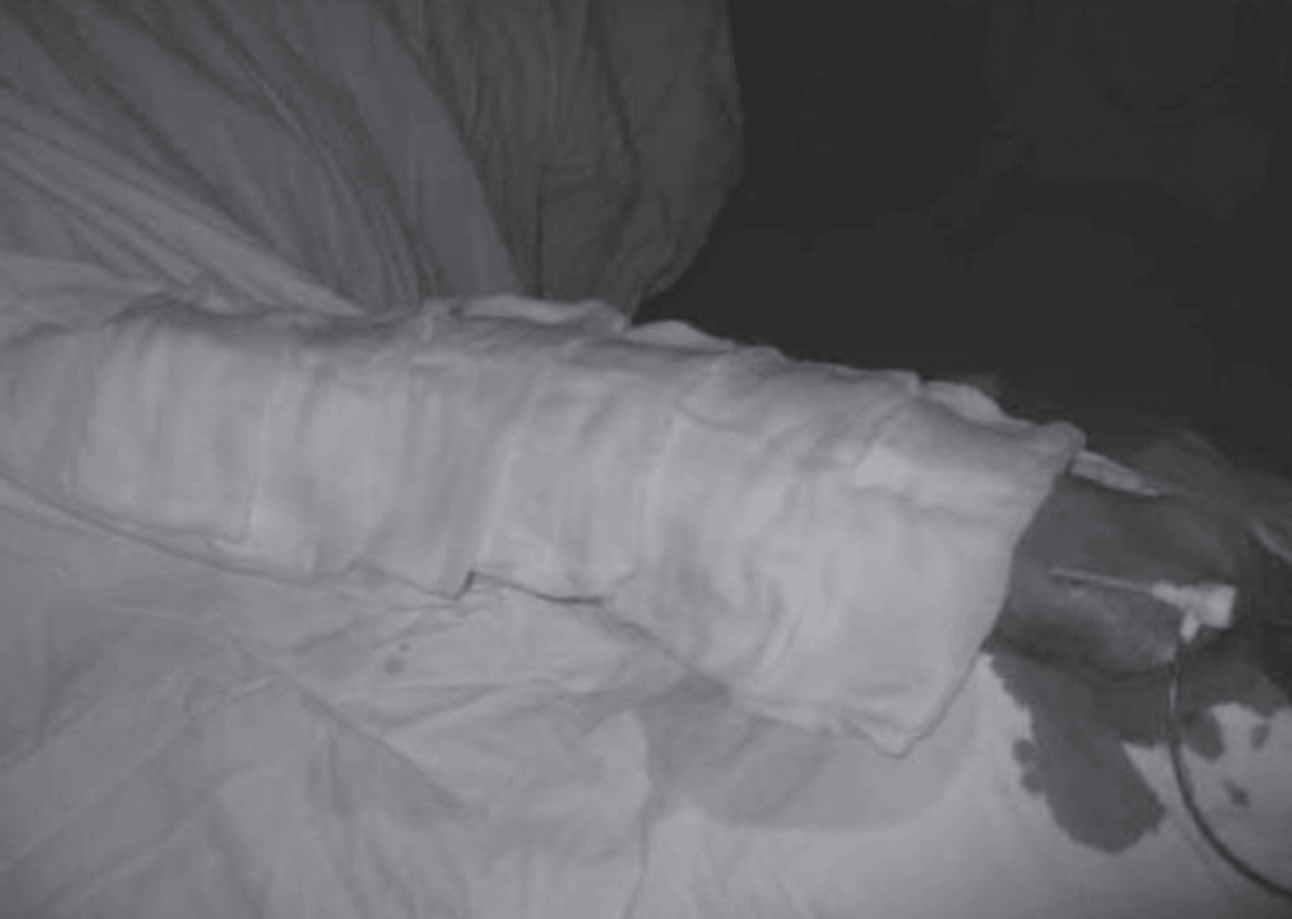
Antecubital surface of the forearm and the arm warming technique with warm towels or surgical gauzes soaked in warm water Reproduced with permission from Zencirci et al., 2016 [10].

**Figure 3:**
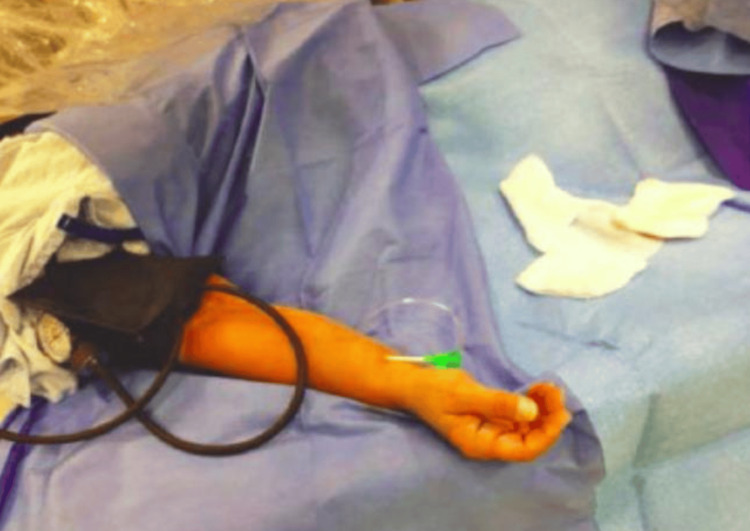
Blood pressure cuff application causing brachial artery occlusion by inflating the cuff 50mmHg above the systolic blood pressure Reproduced with permission from Zencirci et al., 2016 [10].

SCAI guidelines recommend the following four stepwise approaches to remove the entrapped radial artery sheath [12-14]. In the first step, vasodilators, such as verapamil (2.5 to 5 mg), nitroglycerin (200-600 mcg), and additional sedation, such as opioid and benzodiazepine, can also be considered. The next step is using forearm warming techniques with an air patient warming system for up to 15 minutes at 43°C or covering the forearm's antecubital surface and arm with warm towels soaked in warm water at a temperature of approximately 50 °C. If this technique also proves to be ineffective, the next step is to apply the blood pressure cuff to the upper forearm for 5 min by inflating it 40 mmHg above the systolic blood pressure to occlude the brachial artery. This is also known as the "clamp-and-release" technique, and can lead to a potent smooth muscle relaxation response, facilitating radial sheath removal. If the above techniques fail, then deep sedation or general anaesthesia should be considered to decrease the neurogenic influence The final step is regional nerve block or surgical endarterectomy as a last resort in patients who do not respond to any of the above treatments (Figure 1-3).

## Conclusions

In conclusion, severe radial artery spasms resulting in radial artery sheath entrapment is a rare complication, and intervention cardiologists should be familiar with techniques for sheath removal. Entrapped radial artery sheaths should not be pulled out as this can lead to radial artery avulsion, necessitating that patients undergo endarterectomy. Several techniques can be used to successfully remove the entrapped radial artery sheaths, including the use of sedatives, nitrates, radial cocktail and verapamil, clamp-and-release techinque, and deep sedation. This leads to reduced anxiety, stress, and vasodilatation, thus reducing the risk of radial artery spasm. Our case report highlights the significance of following a stepwise approach for removing entrapped radial artery sheaths.

## References

[1] Curtis E, Fernandez R, Khoo J, Weaver J, Lee A, Halcomb E (2023). Clinical predictors and management for radial artery spasm: an Australian cross‑sectional study. BMC Cardiovasc Disord.

[2] Feldman DN, Swaminathan RV, Kaltenbach LA (2013). Adoption of radial access and comparison of outcomes to femoral access in percutaneous coronary intervention: an updated report from the national cardiovascular data registry (2007-2012). Circulation.

[3] Rigattieri S, Valsecchi O, Sciahbasi A (2017). Current practice of transradial approach for coronary procedures: A survey by the Italian Society of Interventional Cardiology (SICI-GISE) and the Italian Radial Club. Cardiovasc Revasc Med.

[4] Hamon M, Pristipino C, Di Mario C (2013). Consensus document on the radial approach in percutaneous cardiovascular interventions: position paper by the European Association of Percutaneous Cardiovascular Interventions and Working Groups on Acute Cardiac Care** and Thrombosis of the European Society of Cardiology. EuroIntervention.

[5] Kolkailah AA, Alreshq RS, Muhammed AM, Zahran ME, Anas El-Wegoud M, Nabhan AF (2018). Transradial versus transfemoral approach for diagnostic coronary angiography and percutaneous coronary intervention in people with coronary artery disease. Cochrane Database Syst Rev.

[6] Giannopoulos G, Raisakis K, Synetos A (2015). A predictive score of radial artery spasm in patients undergoing transradial percutaneous coronary intervention. Int J Cardiol.

[7] Zus AS, Crișan S, Luca S (2024). Radial artery spasm: a review on incidence, prevention and treatment. Diagnostics (Basel).

[8] Ercan S, Unal A, Altunbas G, Kaya H, Davutoglu V, Yuce M, Ozer O (2014). Anxiety score as a risk factor for radial artery vasospasm during radial interventions: a pilot study. Angiology.

[9] Alkhouli M, Cohen HA, Bashir R (2015). Radial artery avulsion--a rare complication of transradial catheterization. Catheter Cardiovasc Interv.

[10] Zencirci E, Değirmencioğlu A (2016). Catheter entrapment due to severe radial artery spasm during transradial approach. Cardiol J.

[11] Schenke K, Viertel A, Joghetaei N (2021). Distal transradial access for coronary angiography and interventions in everyday practice: data from the TRIANGLE registry (TwitteR Initiated registry for coronary ANgiography in Germany via distaL radial accEss). Cardiol Ther.

[12] (2024). The entrapped radial sheath: a stepwise approach algorithm. https://scai.org/entrapped-radial-sheath-stepwise-approach-algorithm.

[13] Barçin C, Kurşaklioğlu H, Köse S, Amasyali B, Işik E (2010). Resistant radial artery spasm during coronary angiography via radial approach responded to local warm compress. Anadolu Kardiyol Derg.

[14] Pancholy SB, Karuparthi PR, Gulati R (2015). A novel nonpharmacologic technique to remove entrapped radial sheath. Catheter Cardiovasc Interv.

